# Sintering Behavior of Molybdenite Concentrate During Oxidation Roasting Process in Air Atmosphere: Influences of Roasting Temperature and K Content

**DOI:** 10.3390/molecules29215183

**Published:** 2024-11-02

**Authors:** Jiangang Liu, Lu Wang, Guohuan Wu

**Affiliations:** 1 College of Mechanical and Electrical Engineering, Wuyi University, Wuyishan 354300, China; liujiangang87@126.com; 2 Key Laboratory for Ferrous Metallurgy and Resources Utilization of Ministry of Education, Wuhan University of Science and Technology, Wuhan 430081, China; 3 School of Intelligent Manufacturing, Wenzhou Polytechnic, Wenzhou 325035, China

**Keywords:** molybdenite concentrate, oxidation roasting, sintering behavior, temperature, K content

## Abstract

Sintering is a common phenomenon, which often takes place during the oxidation roasting process of molybdenite concentrate in multiple-hearth furnaces. The occurrence of sintering phenomena has detrimental effects on the product quality and the service life of the furnace. In this work, the influence of two key factors (roasting temperature and K content) on the sintering behavior is investigated using molybdenite concentrate as the raw material. Different technologies such as XRD, FESEM-EDS, and phase diagrams are adopted to analyze the experimental data. The results show that the higher the roasting temperature is, the greater the mass loss and the more serious the sintering degree will be. The results also show that with the increase in K content, the mass loss of the raw material is first increased and then decreased, while its sintering degree is still gradually increased. The sintering products obtained during the oxidation roasting process are often tightly combined with the bottom of the used crucible with a smooth and dense surface structure, while their internal microstructures are very complicated, which not only includes numerous MoO_3_ species, but also unoxidized MoS_2_, Mo sub-oxide, SiO_2_, and a variety of molybdates. Among them, both MoO_3_ and molybdates can be easily dissolved into the ammonia solution, leading to a residue mainly composed of SiO_2_ and CaMoO_4_. This study also finds that the sintering phenomenon is caused by the increase in local temperature and the formation of various low-melting-point eutectics. It is suggested that decreasing the roasting temperature and K content, especially the K content, are effective methods for reducing the sintering degree of molybdenite concentrate during the oxidation roasting process.

## 1. Introduction

The oxidation roasting of molybdenite concentrate in multiple-hearth furnaces is an important process for preparing molybdenum calcine and the subsequent molybdenum products [1,2,3,4,5,6,7]. Data in the literature show that this process is composed of two main steps: the oxidation of MoS_2_ to MoO_2_ (Reaction (1)) and the further oxidation of MoO_2_ to MoO_3_ (Reaction (2)) [8,9,10,11]. However, the sintering phenomenon always occurs during the process, which makes the prepared molybdenum calcine become dense and hard. The occurrence of the sintering phenomenon will also cause some other adverse effects, such as the incomplete oxidation of molybdenite concentrate and the increase in residual sulfur content. In addition, the target teeth and target arm of the multiple-hearth furnace may be damaged if the sintering phenomenon is serious enough, which will even lead to the occurrence of a “dead furnace”. In order to enhance the product quality of molybdenum calcine and improve the service life of multiple-hearth furnaces, a systematic analysis of the sintering mechanism, therefore, is important.

MoS_2_ + 3O_2_ = MoO_2_ + 2SO_2_ (1)

MoO_2_ + 0.5O_2_ = MoO_3_ (2)

Numerous papers about the sintering behaviors of molybdenite concentrate have been reported [12,13], and they found that the main influencing factors include roasting temperature, impurity type and content, sample thickness, stirring speed, reaction time, oxidation atmosphere and pressure, etc. [14,15,16,17]. For example, our previous work [18] demonstrated that sintering phenomena will not occur when adopting air as the oxidation atmosphere at 600 °C, whereas when oxygen is adopted, the sintering phenomenon will occur. The influence of different factors (roasting temperature, impurity type, and content) on the sintering degree of pure MoS_2_ were also investigated [19], and the results showed that impurities such as elements Al and Si had no obvious effect on the sintering behavior; as for the impurities such as elements Ca, Fe, Pb, Mg, Cu, and K, however, the effects were serious. Similar results were also reported by Bu et al. [20], in which molybdenite concentrate was used as the raw material.

Even though many valuable conclusions about the sintering behavior have been drawn in recent decades, some issues still exist. For example, research concerning the influence of different factors on the regular phase transition of the sintering product is lacking; as to the morphology evolution behavior, related research is also scarce. In order to make up for these gaps, the current work was initiated. On one hand, K impurities were reported to have serious influences on the sintering behavior [19]; on the other hand, it was considered as a harmful element on the product performance of Mo-based alloys [21,22]. Therefore, K impurities were selected as the main study object in this work. In addition, the influence of roasting temperature on the sintering behavior of molybdenite concentrate during the oxidation roasting process was also investigated.

## 2. Results

### 2.1. Mass Loss and Sintering Degree

The mass loss of the mixed sample and the mass of the sintering product were calculated by the following equations:

(3) $$m_{\mathrm{mass}\;\mathrm{loss}}=m_{\mathrm{total}}-m_{\mathrm{residual}\;\mathrm{sample}}$$

(4) $$m_{\sin\mathrm{tering}\;\mathrm{product}}=m_{\mathrm{residual}\;\mathrm{sample}}-m_{\mathrm{surface}\;\mathrm{loose}}$$

where $m_{\mathrm{mass}\;\mathrm{loss}}$ is the mass loss of the mixed sample, g; $m_{\mathrm{residual}\;\mathrm{sample}}$ and $m_{\mathrm{surface}\;\mathrm{loose}}$ are the masses of the residual sample and the surface loss sample after the roasting reaction, respectively, g; *m*_total_ is the initial mass of the used mixed sample; $m_{\sin\mathrm{tering}\;\mathrm{product}}$ is the mass of the sintering product that adhered tightly to the bottom of crucible.

The sintering degree of the used mixed sample was defined as the ratio of the sintering product mass to the residual sample mass, see Equation (5):

(5) $$\beta =\frac{m_{\sin\mathrm{tering}\;\mathrm{product}}}{m_{\mathrm{residual}\;\mathrm{sample}}}$$

#### 2.1.1. Influence of Roasting Temperature

**Figure 1:**
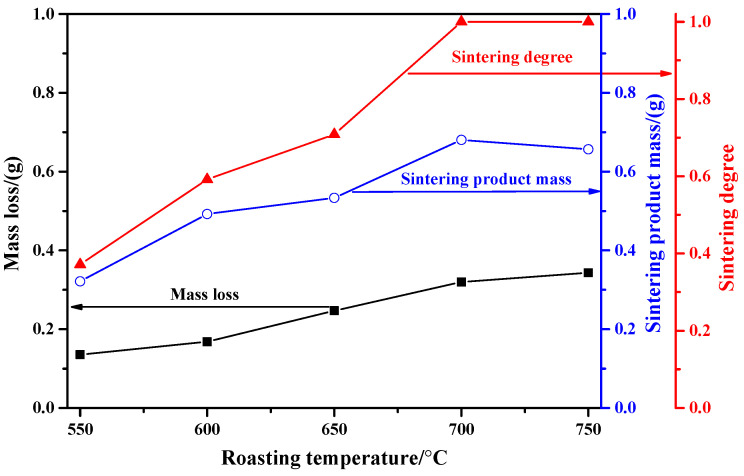
Influence of roasting temperature on the mass loss and sintering degree of the used mixed sample (K content: 2%).

Figure 1 shows the influence of roasting temperature on the mass loss and sintering degree of the used mixed sample. When the roasting temperature is 550 °C, the mass loss and sintering product mass are both small, which may be due to the low roasting temperature and reaction rate. When the roasting temperature increased from 550 °C to 700 °C, the values of mass loss and sintering product mass are both gradually increased. At 700 °C, the sintering product mass reaches its peak value (0.6803 g). When the roasting temperature increased to 750 °C, the mass loss is still increased, while the sintering product mass begins to decrease, which may be due to the strong sublimation effect of MoO_3_ at higher-temperature atmospheres [23,24,25]. As for the sintering degree, the results show that its value is gradually increased until reaching 1 at 700 °C; that is to say, when the roasting temperature is higher than 700 °C, the residual samples are all sintering products and they are all firmly glued to bottom of the crucible.

#### 2.1.2. Influence of K Content

**Figure 2:**
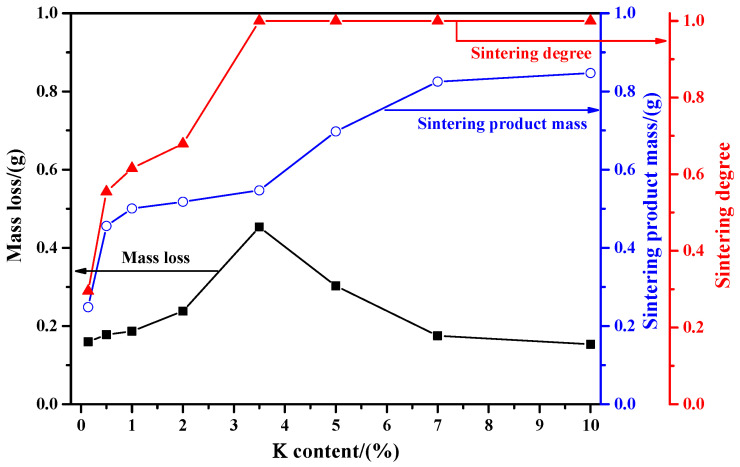
Influence of K content on the mass loss and sintering degree of the used mixed sample (roasting temperature: 650 °C).

Figure 2 shows the influence of K content on the mass loss and sintering degree of the used mixed sample. From this figure, it can be found that K content has significant influences on the mass loss: when the K content is below 1%, the mass loss is gradually increased; however, due to the small amount of K_2_CO_3_, the release rate of gaseous products is relatively slow, and thus the increase rate of mass loss is relatively slow. When the K content is in the range of 1% to 3.5%, the results show that the mass loss is increased with a relatively fast increase rate, which may be due to the higher release rate of gaseous products, especially for SO_2_ and CO_2_. Moreover, the maximum mass loss of 0.4532 g will be reached at a K content of 3.5%. Strangely, when the K content is further increased (above 3.5%), the mass loss begins to decrease. For this phenomenon, we have conducted several trials with the same experimental samples and procedures in order to verify the result, and the obtained mass losses are all similar to those that displayed in Figure 2, which indicates that the displayed results have a good reproducibility. Herein, the reason for the decrease in the mass loss at a higher K content may mainly result from the strong sintering behavior and the incomplete oxidation of molybdenite concentrate. Figure 2 also shows that the sintering product’s mass and sintering degree are both increased with the increasing K content: when the K content is 3.5%, the sintering product mass is 0.5468 g and the sintering degree reaches 1, which indicates that under these conditions the residual samples are all becoming sintering products. Further increasing the K content, the sintering product mass is still increased, and the sintering degree always remains constant at 1. Herein, the ever-increasing sintering product mass is another important reason for the ever-decreasing mass loss under the conditions of a K content above 3.5%.

### 2.2. Phase Composition of Sintering Product

The phase composition of the surface loss sample after the roasting reaction is first checked, and the result shows that it is mainly composed of MoO_3_; in addition, its morphological structure is observed, and the results demonstrate that it will transform from an irregular granular structure to a platelet-shaped structure as the roasting temperature increases. The results agree well with our previous work [18]. However, since the main task of this work is to investigate the influence of roasting temperature and K content on the sintering behavior of molybdenite concentrate during the oxidation roasting process, the systematic analysis of the two factors on the phase composition of the sintering product is therefore the focus.

#### 2.2.1. Influence of Roasting Temperature

**Figure 3:**
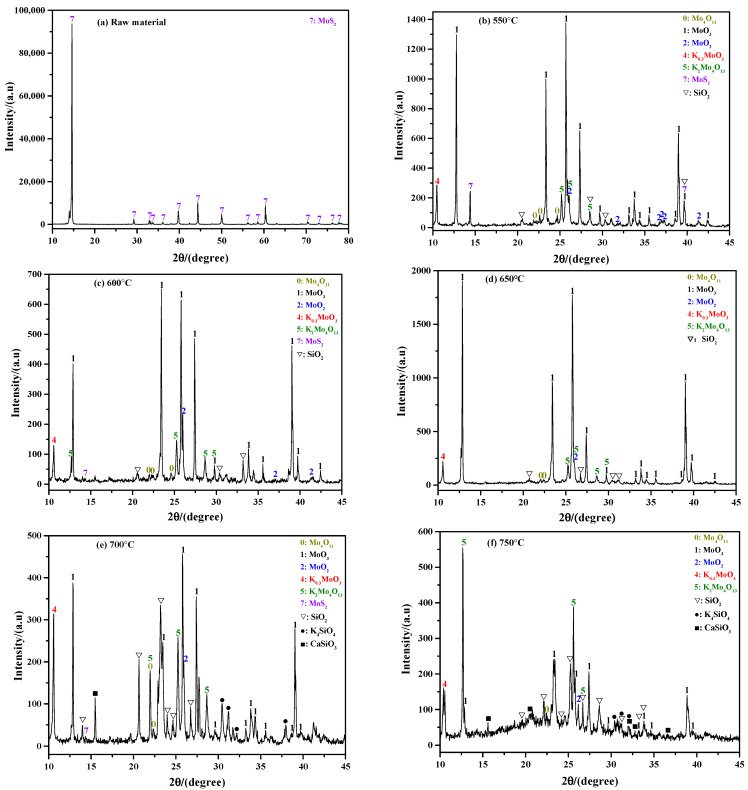
XRD patterns of sintering product obtained at different roasting temperatures: (**a**) raw material; (**b**) 550 °C; (**c**) 600 °C; (**d**) 650 °C; (**e**) 700 °C; (**f**) 750 °C. K content: 2%.

Figure 3 shows the influence of roasting temperature on the phase composition of the sintering product. As observed in Figure 3a, the raw material before the roasting reaction is only MoS_2_, and the other impurity components are not detected due to their low amount. When the roasting temperature is 550 °C, the result shows that the sintering product is mainly composed of MoO_3_; in addition, small amounts of MoS_2_, MoO_2_, Mo_4_O_11_, K_0_._3_MoO_3_, K_2_MoO_4_O_13_, and SiO_2_ also exist (see Figure 3b). Increasing the roasting temperature to 600 °C, the result is similar to that obtained at 550 °C; however, the intensity of the diffraction peak of MoS_2_ is greatly weakened (see Figure 3c), indicating most of the MoS_2_ is oxidized. No diffraction peak of MoS_2_ is detected when the roasting temperature increases to 650 °C. This suggests that all the MoS_2_ has been completely oxidized or there is little left; even so, the sintering product is still not only composed of MoO_3_, but also some molybdenum sub-oxides and molybdates (see Figure 3d). When the roasting temperature is 700 °C, the sintering product not only includes all the phases that existed at low temperatures, but some new phases such as K_4_SiO_4_ and CaSiO_3_ are also observed (see Figure 3e). The appearance of the new phases may be due to the complicated chemical reactions that only occur at high temperatures. Figure 3e also shows that the intensity of the diffraction peak of SiO_2_ is much stronger than that observed at low temperatures, meaning its relative amount is increased. Further increasing the roasting temperature to 750 °C, it can be seen that the intensity of the diffraction peak of MoO_3_ is weakened, while that of K_2_MoO_4_O_13_ is enhanced (see Figure 3f). This result indicates that the amount of MoO_3_ is decreased and that of K_2_Mo_4_O_13_ is increased. The reasons for this phenomenon may be due to the strong sublimation effect of MoO_3_ and the favorable formation conditions for K_2_Mo_4_O_13_.

#### 2.2.2. Influence of K Content

**Figure 4:**
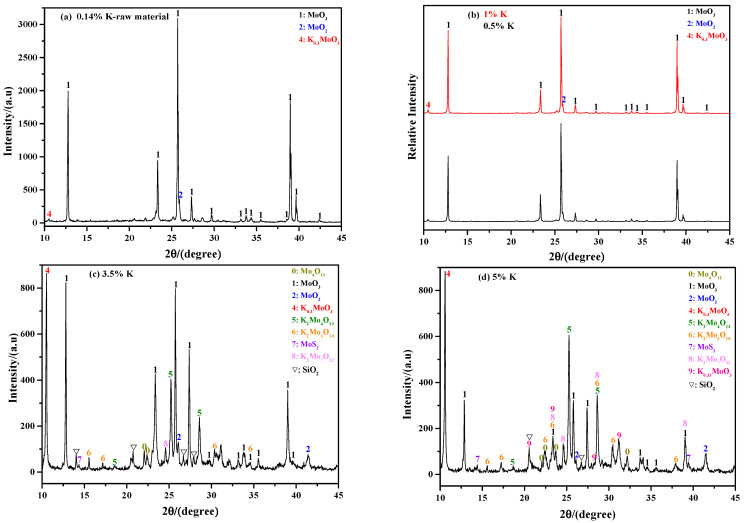

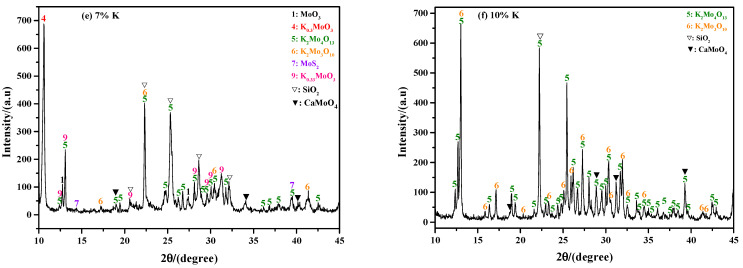
XRD patterns of sintering product obtained at different K contents: (**a**) *ε*_K_ = 0.14%, raw material; (**b**) *ε*_K_ = 0.5% and 1%; (**c**) *ε*_K_ = 3.5%; (**d**) *ε*_K_ = 5%; (**e**) *ε*_K_ = 7%; (**f**) *ε*_K_ = 10%. Roasting temperature: 650 °C.

Figure 4 shows the influence of the K content on the phase composition of the sintering product. When the raw material is used (*ε*_K_ = 0.14%), the results show that the sintering product is mainly MoO_3_ (see Figure 4a); however, a small amount of other phases such as MoO_2_ and K_0_._3_MoO_3_ also exist. The results for the case with *ε*_K_ = 0.5% and 1% are nearly the same as the raw material (see Figure 4b). When the K content increases to 2% (*ε*_K_ = 2%), the result begins to differ, in which the sintering product not only includes all the phases that are observed at low K contents, but some new phases such as K_2_Mo_4_O_13_ and SiO_2_ are also detected, as shown in Figure 3d. Further increasing the K content to 3.5% (*ε*_K_ = 3.5%), the phase compositions become more complicated: on one hand, the amount of K_2_Mo_4_O_13_ is significantly increased; on the other hand, some new molybdates such as K_2_Mo_3_O_10_ and K_2_Mo_7_O_22_ appear (see Figure 4c). When the K content is 5% (*ε*_K_ = 5%), the result is similar to that obtained at *ε*_K_ = 3.5%; however, it can also be found that the relative intensity of the MoO_3_ diffraction peak is significantly decreased, while that of K_2_Mo_4_O_13_ is increased (see Figure 4d); this indicates that the relative amount of MoO_3_ in the sintering product is decreased, while that of K_2_Mo_4_O_13_ increased. The above phenomena become more obvious at a higher K content, where MoO_3_ could even disappears at *ε*_K_ = 10% (see Figure 4e,f). In addition to molybdates, a large amount of SiO_2_ and CaMoO_4_ are also observed at the higher K content conditions, which indicates that the impurities have been enriched at this moment.

### 2.3. Morphological Structure of Sintering Product

#### 2.3.1. Influence of Roasting Temperature

**Figure 5:**
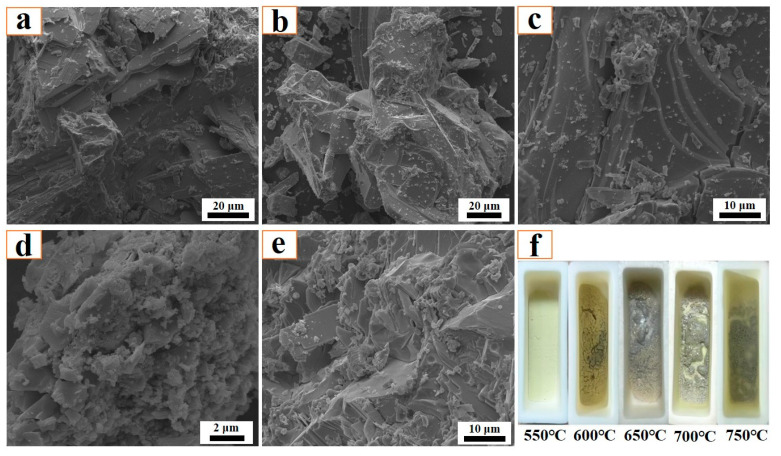
Morphological structure of sintering product obtained at different roasting temperatures: (**a**) 550 °C; (**b**) 600 °C; (**c**) 650 °C; (**d**) 700 °C; (**e**) 750 °C; (**f**) Macroscopic image of the residual sample. K content: 2%.

Figure 5 shows the influence of roasting temperature on the morphological structure of the sintering product. From this figure, it can be observed that roasting temperature has no obvious influence on the microstructure, and all of products exhibit a smooth and dense surface structure. The XRD results in Figure 3 show that the main phase composition of the sintering product is MoO_3_, so it can be deduced that the smooth surface particles are MoO_3_. Figure 5f is the macroscopic image of the residual sample after the roasting reaction, from which it can be observed that when the roasting temperature is low (550 °C and 600 °C), the residual sample exhibits a golden-yellow color and its surface is relatively loose. When the roasting temperature increases to 650 °C and 700 °C, the results show that the residual sample becomes a light grey color, and a smooth and dense surface structure formed by liquid solidification is clearly observed; in this case, the residual sample is hard to remove from the bottom of the crucible. When the roasting temperature increases to 750 °C, the results show that the mass of the residual sample is very small due to the strong sublimation effect of MoO_3_, and no obvious smooth structure is observed, while the residual sample is still tightly bound to the bottom of the crucible.

#### 2.3.2. Influence of K Content

**Figure 6:**
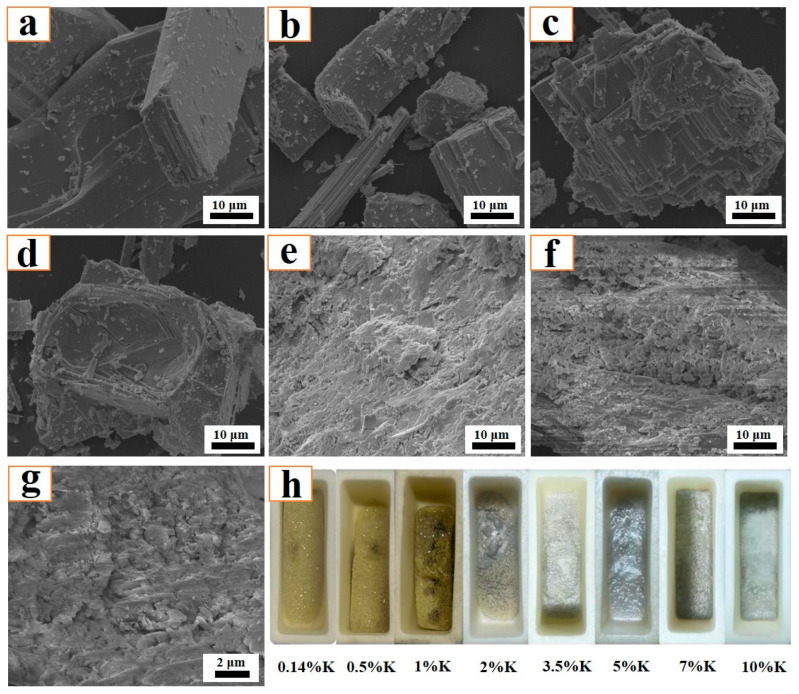
Morphological structure of sintering product obtained at different K contents: (**a**) *ε*_K_ = 0.14%, raw material; (**b**) *ε*_K_ = 0.5%; (**c**) *ε*_K_ = 1%; (**d**) *ε*_K_ = 2%; (**e**) *ε*_K_ = 5%; (**f**) *ε*_K_ = 7%; (**g**) *ε*_K_ = 10%; (**h**) Macroscopic image of the residual sample. Roasting temperature: 650 °C.

Figure 6 shows the influence of K content on the morphological structure of the sintering product. Unlike the influence of roasting temperature, Figure 6 demonstrates that the K content has a certain influence on the microscopic structure. When the K content is relatively low (0.14–2%), the results show that the sintering product mainly exhibits a platelet-shaped structure with a large dimension. Combining the XRD results and other references [18], the platelet-shaped product can be deduced to be MoO_3_. Increasing the K content to a relatively high value (5–10%), the sintering product becomes coarse and the amount of platelet-shaped particles is relatively small, which indicates that the amount of MoO_3_ is decreased. The above results agree well with the XRD results shown in Figure 4.

Figure 6h is a macroscopic image of the residual sample after the roasting reaction. It shows that the residual sample exhibits a golden-yellow color when the K content is below 3.5%, while the surface structure is smoother and denser when the K content is 2% and 3.5%, indicating a large amount of liquid has formed. When the K content increases to 5%, the product’s surface appears grey, with the grey color becoming lighter when the K content is further increased (7–10%).

#### 2.3.3. Cross-Section Microstructure

**Figure 7:**
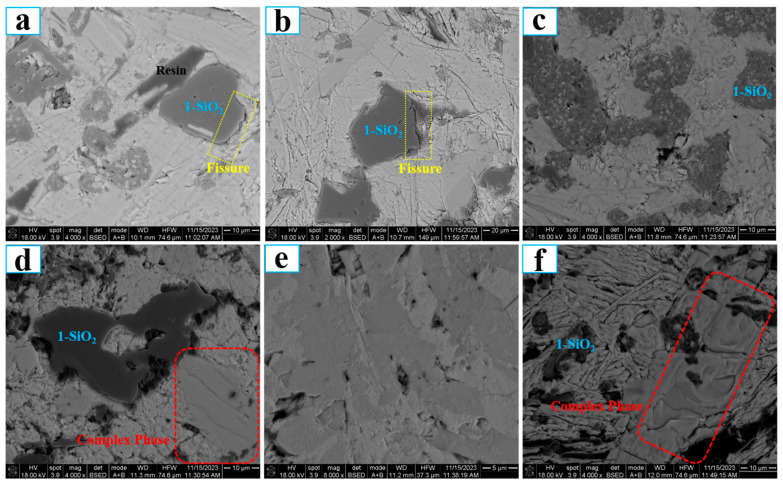
FESEM backscattering micrographs of the cross-section of the sintering product obtained under different conditions: (**a**) 650 °C, *ε*_K_ = 2%; (**b**) 650 °C, *ε*_K_ = 5%; (**c**) 650 °C, *ε*_K_ = 7%; (**d**) 650 °C, *ε*_K_ = 10%; (**e**) 650 °C, *ε*_K_ = 10%; (**f**) 750 °C, *ε*_K_ = 2%.

Both Figure 5 and Figure 6 show the three-dimensional microstructure of the sintering product, and the results are beneficial to the qualitative analysis of the sintering degree. To gain insight into the sintering mechanism of molybdenite concentrate, the cross-section microstructure of the sintering product is also analyzed. Herein, the sintering products obtained under the following conditions are selected as the experimental samples: 650 °C and 2% K, 650 °C and 5% K, 650 °C and 7% K, 650 °C and 10% K, as well as 750 °C and 2% K. The corresponding results are displayed in Figure 7. It shows that all the samples reveal various color regions, such as black, light-grey, and light-white, which indicates that the phase composition of the sintering product is complex. From Figure 7, it can also observe that the black phase (which is identified to be SiO_2_) is not closely connected with the other phases and many cracks/fissures (yellow square area) exist, while the light-grey phase and light-white phase are tightly bonded. The reason for the phenomenon may be due to the different wettabilities between different phases.

**Figure 8:**
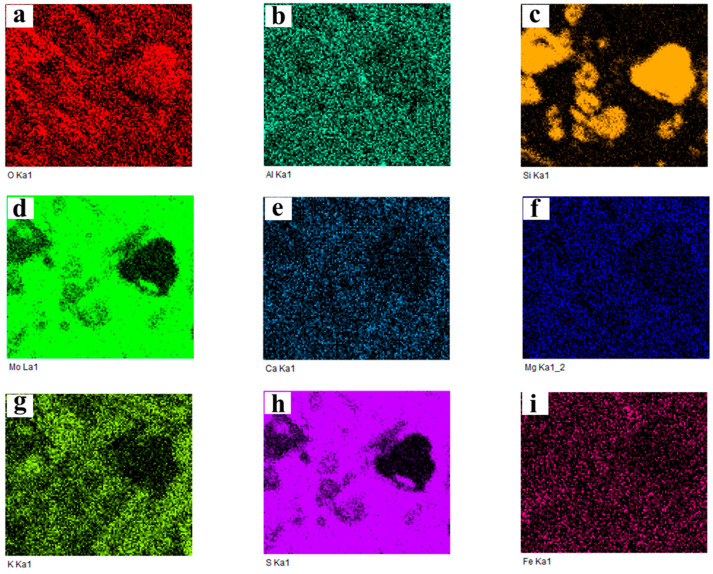
Map scanning result of the cross-section of the sintering product shown in Figure 7a: (**a**) O; (**b**) Al; (**c**) Si; (**d**) Mo; (**e**) Ca; (**f**) Mg; (**g**) K; (**h**) S; (**i**) Fe. (650 °C, *ε*_K_ = 2%).

**Figure 9:**
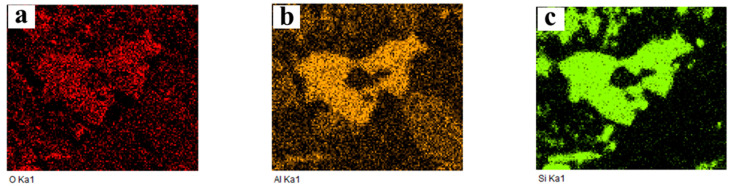

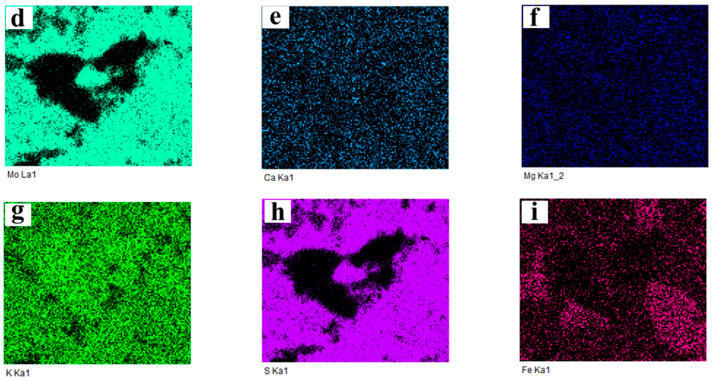
Map scanning result of the cross-section of the sintering product shown in Figure 7d: (**a**) O; (**b**) Al; (**c**) Si; (**d**) Mo; (**e**) Ca; (**f**) Mg; (**g**) K; (**h**) S; (**i**) Fe. (650 °C, *ε*_K_ = 10%).

**Figure 10:**
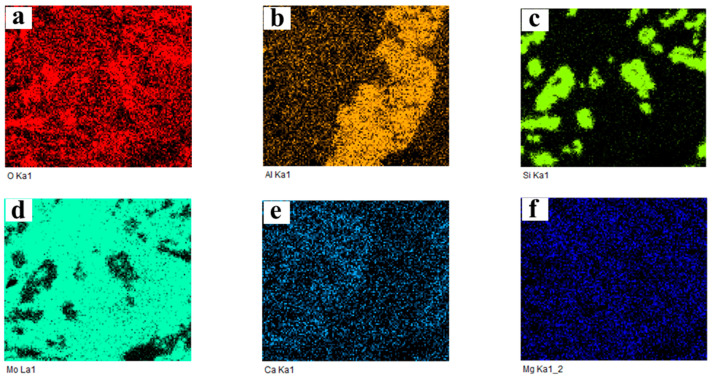

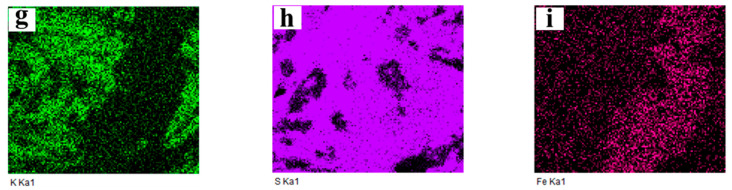
Map scanning result of the cross-section of the sintering product shown in Figure 7f: (**a**) O; (**b**) Al; (**c**) Si; (**d**) Mo; (**e**) Ca; (**f**) Mg; (**g**) K; (**h**) S; (**i**) Fe. (750 °C, *ε*_K_ = 2%).

In order to identify the elemental distribution and possible phase composition of different color areas, EDS area scanning maps are used. In this section, sintering products obtained under the following conditions were selected as the experimental samples: 650 °C and 2% K, 650 °C and 10% K, as well as 750 °C and 2% K. The corresponding map scanning results are presented in Figure 8, Figure 9 and Figure 10, respectively. In the case of 650 °C and 2% K, Figure 8 shows that the main elements are O and Si in the black area, indicating the existence of SiO_2_. The oxidation roasting of molybdenite concentrate in air/oxygen atmosphere was investigated in our previous work [18], and the results found that SiO_2_ also existed in the molybdenum calcine. If the molybdenum calcine contains SiO_2_, undoubtedly, the SiO_2_ will also remain in the sintering product. As for the light-grey and light-white areas, Figure 8 shows that the main elements are Mo and S, indicating the existence of unreacted MoS_2_; furthermore, partial K, Ca, and Mg are also detected in the two regions. Al and Fe are uniformly distributed in the whole field of view. With the increase in the K content to 10%, the main elements in the black area are O, Si, and Al (see Figure 9). The appearance of the additional Al element may be due to the complicated chemical reaction that occurred under the higher K content condition. Some overlapping areas between elements, namely Al and Fe, are also observed (marked by red squares in Figure 7d), indicating that the two elements could combine with each other to form a eutectic. In the case of 750 °C and 2% K, the results show that the phase of the black region is SiO_2_, and Al and Fe also have some overlapping regions (see Figure 10), which is similar to the results shown in Figure 9. However, in this case, elements Al and Si have no obvious overlapping regions. According to the above results, it can be inferred that a higher K content is the prerequisite for the mutual solution of Al and Si; the above results also suggest that the K content has a more significant influence on the sintering behavior than roasting temperature. However, the elements of Al, Fe, and Mg are not detected in the XRD patterns, which may be due to their low amounts, which can even be below the detection limits of XRD measurement.

## 3. Discussion

### 3.1. Ammonia Leaching of Sintering Product

As mentioned above, the phase composition of the sintering product is very complicated, as it not only includes various molybdenum oxides, but also unreacted MoS_2_ and other impurities. To analyze the sintering product and illustrate the sintering mechanism in detail, an ammonia leaching treatment is conducted. The treating process is described as follows: first, a certain amount of sintering product is mixed with 20 mL ammonia solution, with a stirring time of 2 h; second, the mixed solution is filtered by a vacuum filter and washed with deionized water and anhydrous ethanol several times; then, the leaching residue is dried at 110 °C in a drying oven for 8 h; finally, the masses of the used sintering product and obtained ammonia leaching residue are recorded, respectively. After that, the leaching rate (defined as the ratio of the leached mass to the initial mass of used sintering product) of the sintering product is calculated.

#### 3.1.1. Influence of Roasting Temperature

**Figure 11:**
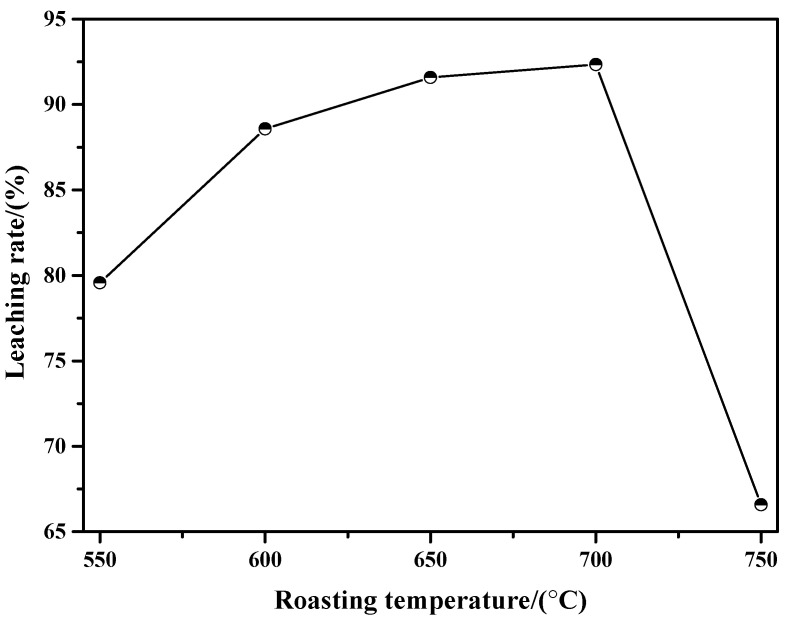
Results of the ammonia leaching rate of sintering product obtained at different roasting temperatures.

Figure 11 shows the ammonia leaching rates of the sintering products obtained at different roasting temperatures. From this figure, it can be found that the leaching rate is gradually increased with the increase in roasting temperature at a value below 700 °C. Specifically, when the roasting temperatures are 550 °C, 600 °C, 650 °C, and 700 °C, their leaching rates are 79.57%, 88.58%, 91.59%, and 92.34%, respectively. On one hand, both MoO_3_ and partial molybdates can be dissolved in ammonia solution; on the other hand, the amount of MoO_3_ dominates in the sintering product according to the above XRD results, so the value of the leaching rate may be considered as the amount of exiting MoO_3_ (in fact, it is a litter bigger). That is, the higher the roasting temperature is, the larger the amount of existing MoO_3_ will be. However, when the roasting temperature is increased to 750 °C, the leaching rate is decreased to a value of only 66.58%. The XRD results in Figure 3f show that the intensity of the MoO_3_ diffraction peak is extremely weak, which also indicates that the amount of MoO_3_ in the sintering product obtained at 750 °C is relatively small. All in all, the results of the ammonia leaching rates are consistent with the XRD results shown in Figure 3.

#### 3.1.2. Influence of K Content

**Figure 12:**
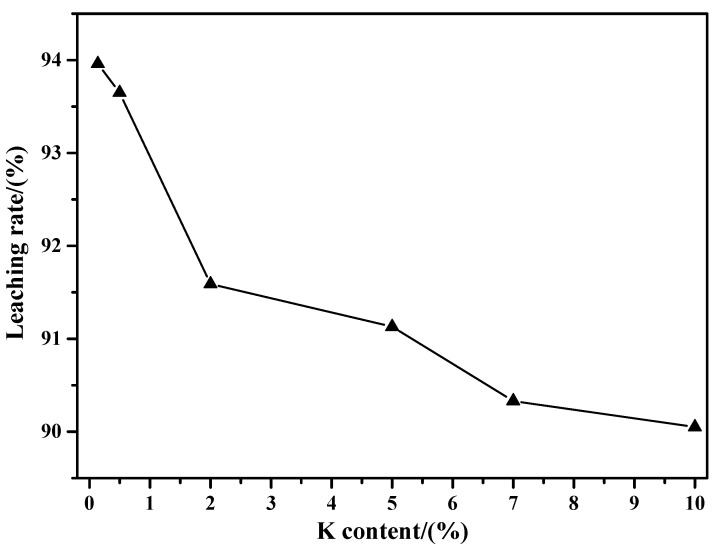
Results of the ammonia leaching rate of sintering products obtained at different K contents.

Figure 12 shows the results of the ammonia leaching rates of the sintering products obtained at different K contents. From this figure, it can be found that the ammonia leaching rate is gradually decreased with the increase in K content. Specifically, when the K contents are 0.14% (raw material), 0.5%, 2%, 5%, 7%, and 10%, the values are 93.96%, 93.65%, 91.59%, 91.13%, 90.33%, and 90.05%, respectively. The results also show that the amount of MoO_3_ in the sintering product is gradually decreased with the increasing K content. The reason for this phenomenon may be due to the rapid formation of insoluble CaMoO_4_ between MoO_3_ and Ca containing impurities. Similarly, the current results agree well with the XRD results shown in Figure 4.

### 3.2. Phase Composition of Ammonia Leaching Residue

#### 3.2.1. Influence of Roasting Temperature

**Figure 13:**
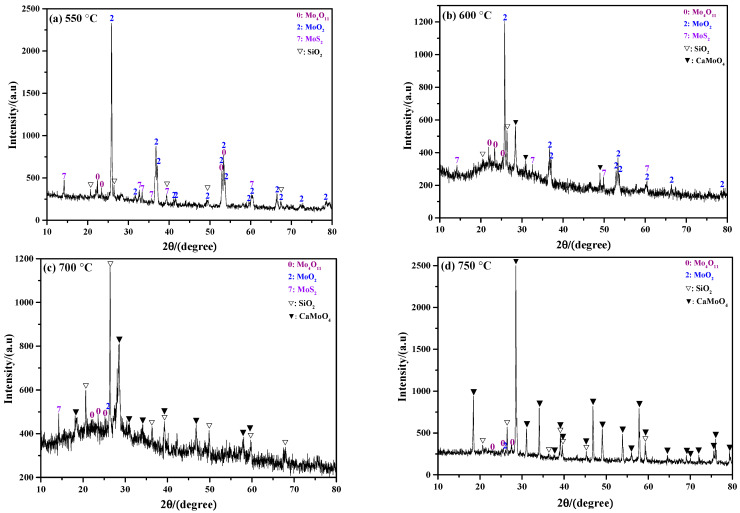
Phase composition of the ammonia leaching residue of sintering products obtained at different roasting temperatures: (**a**) 550 °C; (**b**) 600 °C; (**c**) 700 °C; (**d**) 750 °C. K content: 2%.

Figure 13 shows the phase compositions of the ammonia leaching residues of sintering products obtained at different roasting temperatures. At 550 °C, the XRD results show that the intensity of the MoO_2_ diffraction peak is the strongest, indicating that MoO_2_ is the main component of the ammonia leaching residue; in addition, small amounts of MoS_2_, SiO_2_, and Mo_4_O_11_ are also included (see Figure 13a). When compared with the XRD result in Figure 3b, it is found that the components of MoO_3_, K_0_._3_MoO_3_, and K_2_Mo_4_O_13_ are absent, which indicates that the three components are easily soluble in ammonia solution. When the roasting temperature is 600 °C, the results show that MoO_2_ still has the largest amount, and the components of MoS_2_, SiO_2_, and Mo_4_O_11_ still exist (see Figure 13b). The only difference is that a lot of CaMoO_4_ appears at this temperature. However, the phase of CaMoO_4_ is not detected in the sintering product according to Figure 3c; the reason for this phenomenon may be due to its extremely low amount in the sintering product. The above results also indicate that the ammonia leaching treatment could not only estimate the content of phase that could be dissolved in ammonia solution, but could also enrich and identify potential phases with present in relatively low amounts. When the roasting temperature is 700 °C, the phase composition of the ammonia leaching residue is the same as that obtained at 600 °C, while the main components are transformed into SiO_2_ and CaMoO_4_ instead, which can be clearly deduced from their strong diffraction peak intensities (see Figure 13c). Further increasing the roasting temperature to 750 °C, both SiO_2_ and CaMoO_4_ are still the main components, while the amount of CaMoO_4_ dominates (see Figure 13d). In combination with Figure 3e,f, it can be observed that the phases of K_4_SiO_4_ and CaSiO_3_ disappear, suggesting that both of them have a strong solubility in ammonia solution.

#### 3.2.2. Influence of K Content

**Figure 14:**
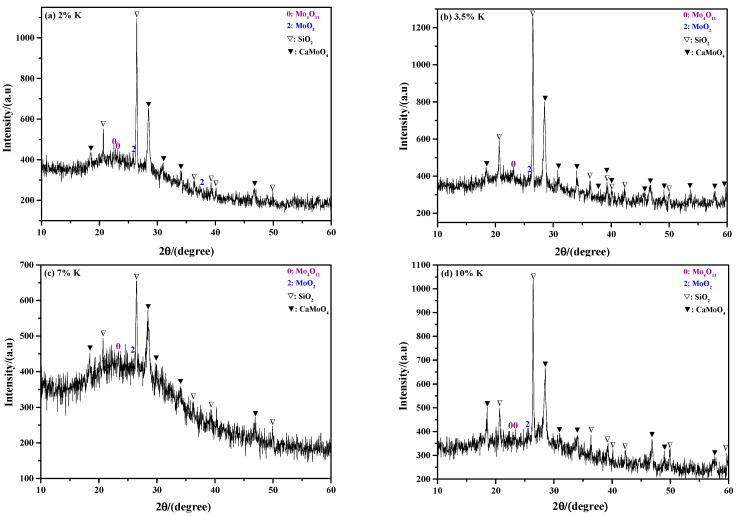
Phase composition of the ammonia leaching residues of sintering products obtained at different K contents: (**a**) 2% K; (**b**) 3.5% K; (**c**) 7% K; (**d**) 10% K. Roasting temperature: 650 °C.

To analyze the influence of K content on the phase composition of ammonia leaching residue, the sintering products obtained at the K contents of 2%, 3.5%, 7%, and 10% were selected as reference materials, and the corresponding XRD results are presented in Figure 14. From the figure, it can be seen that the main phase compositions are SiO_2_, CaMoO_4_, Mo_4_O_11_, and MoO_2_; among them, the amount of SiO_2_ dominates in the ammonia leaching residue. In fact, partial MoS_2_ may also be present, although it is not detected due to its relatively small amount. Combining Figure 13 and Figure 14, it can be concluded that roasting temperature has a more significant influence than K content on the phase composition of the ammonia leaching residue: when the roasting temperatures are in the range of 550–600 °C, 650–700 °C, and above 750 °C, the dominant phases in the ammonia leaching residue are MoO_2_, SiO_2_, and CaMoO_4_, respectively.

### 3.3. Sintering Mechanism Analysis

During the oxidation roasting process of molybdenite concentrate, the higher the roasting temperature is, the faster the formation rate of MoO_3_ will be; thus, there will also be a higher content of MoO_3_ in sintering product. In addition, due to the strong exothermic effect of the oxidation roasting process of molybdenite concentrate, at a higher roasting temperature, more heat will be released, which may lead to a rapid increase in the local reaction temperature. Once the temperature is higher than the melting point of MoO_3_ (795 °C) [26,27,28], the generated MoO_3_ will be melted. The liquid MoO_3_ can adsorb the incompletely oxidized MoS_2_ and other solid impurity particles around it, resulting in the occurrence of the sintering phenomenon. During the sintering process, the incompletely oxidized MoS_2_ can react with MoO_3_ to form MoO_2_ (see Equation (6)) [29,30,31]. This is an important reason for the existence of MoO_2_ in the sintering product. Therefore, in order to reduce the occurrence of the sintering phenomenon, the roasting temperature should not be too high. However, if the roasting temperature is too low, the oxidation rate of molybdenite concentrate will be greatly decreased and then a longer time is required for the completion of the oxidation reaction; In addition, a low roasting temperature can easily lead to the incomplete oxidation of MoS_2_, which will not only reduce the production efficiency, but also increase the amount of residual sulfur in molybdenum calcine. Therefore, the roasting temperature should also not be too low [32]. According to the results of the current work and the practices in industrial production, this work believes that 650 °C is a good choice.

MoS_2_ + 6MoO_3_ = 7MoO_2_ + 2SO_2_ (6)

**Figure 15:**
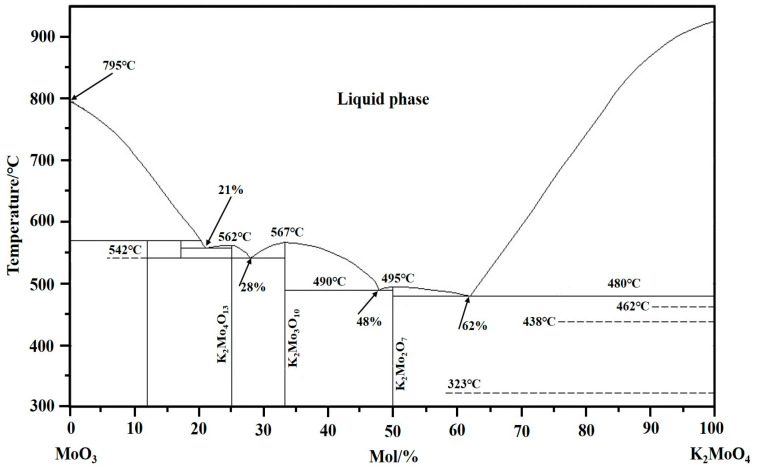
Phase diagram for the K_2_MoO_4_–MoO_3_ binary system [19,20,33].

K_2_CO_3_ is easily decomposed into potassium oxide in a high-temperature environment; in this situation, the reactive activity of the newly formed potassium oxide is relatively high, which will make it easy to react with MoO_3_ to form K_2_MoO_4_ during the oxidation roasting process of molybdenite concentrate. The generated K_2_MoO_4_ may further react with MoO_3_ again to form various eutectics, as can be supported by the binary phase diagram between K_2_MoO_4_ and MoO_3_ see Figure 15 [19,20,33], which shows that the eutectics such as K_2_Mo_4_O_13_ (3MoO_3_·K_2_MoO_4_), K_2_Mo_3_O_10_ (2MoO_3_·K_2_MoO_4_), and K_2_Mo_2_O_7_ (MoO_3_·K_2_MoO_4_) can be formed with the increase in K_2_MoO_4_). Figure 15 also shows that when the contents of K_2_MoO_4_ are 21%, 28%, 48%, and 62%, the melting points of the eutectics are 558 °C, 542 °C, 490 °C, and 480 °C, respectively. These temperatures are not only lower than the melting point of MoO_3_, but also lower than the used experimental temperatures. In other words, K_2_MoO_4_ can form a variety of low-melting-point eutectics with MoO_3_, and their melting points are gradually decreased with the increase in K_2_MoO_4_. Once the above low-melting-point eutectics are formed, they will be rapidly melted within the temperature range of this work. Obviously, their melting speeds are faster than that of MoO_3_. After melting, the liquid low-melting-point eutectics are more likely to entrain the solid particles (MoO_2_, SiO_2_, and MoS_2_, etc.) around them to form complex molten salt mixtures, in which some reactions may occur to produce various silicates (such as K_4_SiO_4_ and CaSiO_3_) and molybdates (such as CaMoO_4_). Therefore, with the increase in K content, the sintering phenomenon will become more and more serious.

In our previous work [19], the influence of K content on the sintering behavior of pure MoS_2_ during the oxidation roasting process was investigated, and the results showed that the sintering degree was 0.6 under the conditions of 650 °C and 2% K. In the current work, the sintering degree is 0.68 under the same temperature and K content conditions, which is higher than the value reported in our previous work. Moreover, the sample mass used in the previous work is 5 g, while that used in the current work is 1 g; that is to say, the sample thickness used in our previous work was thicker than that used in the current work under the same crucible conditions. When the used sample (molybdenite concentrate) has the same thickness as our previous work, Bu’s work [20] found that the sintering degree was up to 0.8 under the same temperature and K content conditions. The results of the current and previous work once again conclude that sample thickness and impurity elements have important influences on the occurrence of sintering phenomena. In addition, the interactions between different impurities (such as Ca, Fe, Pb, Al, etc.) will also occur, and thus a eutectic composed of three or four elements or even more may be formed. This speculation can be confirmed by the images of the cross-section microstructures of the sintering products shown in Figure 7, Figure 8, Figure 9 and Figure 10. The melting point of the multicomponent eutectic may be much lower than that of the binary system. In this case, they are easier to melt, leading to the occurrence of sintering phenomena, which is another reason why the sintering degree is higher when molybdenite concentrate is used as the raw material.

Due to the fact that both SiO_2_ and MoO_3_ are acidic oxides, no reactions between them will occur during the sintering process. In addition, due to the insolubility of SiO_2_ in ammonia solution, the excess Si can exist in the form of SiO_2_ in both the sintering product and the ammonia leaching residue. Ca impurities in the molybdenite concentrate generally exist in the form of alkaline oxide or sulfide, and its sulfide will be oxidized into an oxide during the process, so Ca impurities can combine with MoO_3_ to form CaMoO_4_, which can remain in the ammonia leaching residue due to its insolubility in ammonia solution. The XRD results in Figure 13d show that the relative amount of CaMoO_4_ is higher under the high-temperature conditions, so it can be speculated that increasing the roasting temperature is conducive to the formation of CaMoO_4_. Even if Ca impurities have a certain influence on the sintering behavior of molybdenite concentrate, as reported by [19,20], when compared with K impurities, the degree of influence of Ca is negligible. In fact, it is difficult for us to completely abolish the occurrence of sintering phenomena during the industrial production process; however, decreasing the degree of sintering is still possible. Herein, the work believes that decreasing the roasting temperature and the content of elemental impurities, especially for K, are effective choices.

## 4. Materials and Experimental Procedure

### 4.1. Materials

Molybdenite concentrate from Jinduicheng Molybdenum Industry Co., Ltd., Xi’an, China, was used as the raw material. The main elemental components of molybdenite are Mo and S with respective contents of 54.89% and 33.27%. In addition, other impurities components such as Pb (0.08%), Si (1.83%), Cu (0.07%), Ca (0.28%), Fe (1.12%), Al (0.19%), K (0.14%), Ti (0.04%), C (0.17%), and P (0.01%) are also included. The XRD results show that the raw material is mainly composed of MoS_2_, and the FESEM imaging shows that it has a layered structure with a wide size distribution ranging from several microns to 100 μm [18].

To investigate the influence of K content on the sintering behavior, a small amount of extra K was added into the raw material in the form of K_2_CO_3_. According to the content of K, the mass of the added K_2_CO_3_ was calculated using the following Equation (7):

(7) $$m_{K_{2}{\mathrm{CO}}_{3}}=\frac{M_{K_{2}{\mathrm{CO}}_{3}}}{2M_{K}}\cdot m_{\mathrm{total}}\cdot \varepsilon_{K}$$

where $m_{K_{2}{\mathrm{CO}}_{3}}$ is the mass of added K_2_CO_3_, g; $M_{K_{2}{\mathrm{CO}}_{3}}$ is the relative molecular mass of K_2_CO_3_, g/mol; *M*_K_ is the relative atomic mass of element K, g/mol; $m_{\mathrm{total}}$ is the total mass of molybdenite concentrate and added K_2_CO_3_ (herein, its value is fixed to be 1 g); and *ε*_K_ is the total content of element K, %.

Since the initial K content (0.14%) in molybdenite concentrate is lower than the XRD detection limit, to explore the evolution behavior of K-containing compounds during the roasting process, the K content must be enlarged. Moreover, a higher K content could lead to a larger mass of the sintering product; in this case, weighing errors can be greatly reduced and the sintering degree can be easily evaluated. Therefore, the K content was selected from 1 to 10% in this work. Based on the different K contents, the masses of molybdenite concentrate and added K_2_CO_3_ can be calculated, and the corresponding results are listed in Table 1.

### 4.2. Experimental Procedure

In order to simulate the oxidation roasting process of molybdenite concentrate in multiple-hearth furnace, muffle furnace was selected as the experimental equipment in the work. In each of the experimental run, the mass of molybdenite concentrate and required K_2_CO_3_ were first weighed according to Table 1, and then the mixture of them were put into an agate mortar and grinded for 30 min; after that, the mixed sample was loaded into an alumina crucible with the dimension of 50 mm × 20 mm × 20 mm, and then the sample-containing crucible was put into the center of muffle furnace, the temperature of which is aforehand raised to the desired value. When the sample was oxidized completely (herein, the roasting time was enough, about 3 h), taking out the reaction product and cooling it to room temperature. The masses of the residual sample and the surface loose sample were weighed, respectively. To explore the influence of roasting temperature on the sintering behavior, the mixed sample with the K content of 2% was used as the reference material, and the roasting temperatures were set as 550 °C, 600 °C, 650 °C, 700 °C, and 750 °C.

### 4.3. Characterization Methods

The phase composition of the sintering product was analyzed by X-ray diffraction analyzer (XRD; XPert PRO MPD PANalytical; Netherlands; sweep speed: 10°/min; operation voltage: 30 kV; operation current: 30 mA). The morphological structure of the sintering product was observed by field emission scanning electron microscope (FESEM; Thermo Fisher, Waltham, MA, USA; FEI Corporation, Hillsboro, OR, USA; operation voltage: 18 kV).

## 5. Conclusions

In this work, the influence of roasting temperature and K content on the sintering behavior of molybdenite concentrate during the oxidation roasting process in an air atmosphere was investigated. The following conclusions are drawn:

(1) When the roasting temperature is in the range of 550 °C to 700 °C, the mass loss of the molybdenite concentrate, the mass of the sintering product, and the sintering degree are all increased with the increase in roasting temperature. Meanwhile, when the roasting temperature is above 750 °C, the mass of the sintering product is decreased due to the intense sublimation effect of MoO_3_.
(2) With the increase in K content, the mass loss of molybdenite concentrate is increased first and then gradually decreased. The maximum mass loss is reached at a K content of 3.5% with a sintering degree close to 1. This work also found that the sintering product mass is continuously increased with the increase in K content.
(3) The phase composition of sintering product has a certain relationship with the roasting temperature and K content. In general, the sintering product not only contains a large amount of MoO_3_, but also contains numerous unoxidized MoS_2_, molybdenum sub-oxide, SiO_2_, and various molybdates; among these, most of the MoO_3_ and molybdates can be removed after ammonia leaching treatment.
(4) The occurrence of the sintering phenomenon is due to the increase in local reaction temperature and the formation of various low-melting-point eutectics. This work also finds that decreasing the roasting temperature and K content, especially the K content, are effective strategies to reduce the sintering degree of molybdenite concentrate during the oxidation roasting process.

## Figures and Tables

**Table 1:** The masses of used molybdenite concentrate and added K_2_CO_3_.

Total K Content, *ε*_K_/%	0.14	0.5	1	2	3.5	5	7	10
Mass of molybdenite concentrate/g	1	0.9936	0.9847	0.9670	0.9405	0.9138	0.8783	0.8251
Mass of added K_2_CO_3_/g	0	0.0064	0.0153	0.0330	0.0595	0.0862	0.1217	0.1749

Note: 0.14% is the initial K content in the raw material.

## Data Availability

The raw data can be provided as requested upon reasonable request.
